# Predicting postoperative hypocortisolism in patients with non-aldosterone-producing adrenocortical adenoma: a retrospective single-centre study

**DOI:** 10.1007/s40618-023-02283-1

**Published:** 2024-02-22

**Authors:** I. Bonaventura, A. Tomaselli, F. Angelini, D. Ferrari, D. De Alcubierre, V. Hasenmajer, E. Sbardella, A. Cozzolino, A. M. Paganini, A. M. Isidori, M. Minnetti, R. Pofi

**Affiliations:** 1 Department of Experimental Medicine, Sapienza University of Rome, Rome, Italy; 2 Cancer Research Center of Lyon, Inserm U1052, CNRS UMR5286, Claude Bernard Lyon 1 University, Lyon, France; 3 Department of General Surgery, Surgical Specialties and Organ Transplantation “Paride Stefanini”, Sapienza University of Rome, Rome, Italy; 4 Policlinico Umberto I, Centre for Rare Diseases (Endo-ERN Accredited), Rome, Italy; 5 Oxford Centre for Diabetes, Endocrinology and Metabolism, NIHR Oxford Biomedical Research Centre, University of Oxford, Churchill Hospital, Oxford, UK

**Keywords:** Adrenal incidentaloma, Recovery, Hypothalamus–pituitary–adrenal axis, Mild autonomous cortisol secretion, Non-aldosterone-producing adrenocortical adenoma, Postoperative hypocortisolism

## Abstract

**Background:**

Limited information exists on postoperative hypocortisolism and hypothalamus–pituitary–adrenal axis recovery in patients with adrenal incidentaloma following unilateral adrenalectomy. We evaluated frequency of postoperative hypocortisolism and predictors for recovery in non-aldosterone-producing adrenocortical adenoma patients after unilateral adrenalectomy.

**Methods:**

A retrospective analysis of 32 adrenal incidentaloma patients originally included in the ITACA trial (NCT04127552) with confirmed non-aldosterone-producing adrenocortical adenoma undergoing unilateral adrenalectomy from September 2019 to April 2023 was conducted. Preoperative assessments included adrenal MRI, anthropometrics, evaluation of comorbidities, adrenal function assessed via ACTH, urinary free cortisol, and 1 mg dexamethasone suppression test. ACTH and serum cortisol or Short Synacthen test were performed within 6 days, 6 weeks, 6 months, and a year after surgery.

**Results:**

Six days postoperative, 18.8% of patients had normal adrenal function. Among those with postoperative hypocortisolism, 53.8% recovered by 6 weeks. Patients with earlier adrenal recovery (6 weeks) had lower preoperative 1 mg dexamethasone suppression test (median 1 mg dexamethasone suppression test 76.2 [61.8–111.0] nmol/L vs 260.0 [113.0–288.5] nmol/L, *p* < 0.001). Univariate analysis showed preoperative 1 mg dexamethasone suppression test negatively related with baseline ACTH levels (*r* = − 0.376; *p* = 0.041) and negatively associated with the 6-week baseline (*r* = − 0.395, *p* = 0.034) and 30-min cortisol levels during Short Synacthen test (*r* = − 0.534, *p* = 0.023). Logistic regression analysis demonstrated preoperative 1 mg dexamethasone suppression test as the only biochemical predictor for 6-week adrenal recovery: ROC curve identified a 1 mg dexamethasone suppression test threshold of 131 nmol/L predicting 6-week recovery with 89.5% sensitivity and 72.7% specificity (AUC 0.87; 95% CI 66.9–98.7, *p* < 0.001). Other preoperative assessments (tumor size, ACTH levels and anthropometrics) were not associated with postoperative hypothalamus–pituitary–adrenal axis function, but the presence of diabetes was associated with a lower probability of recovery (OR = 24.55, *p* = 0.036). ACTH levels increased postoperatively in all patients but did not predict hypothalamus–pituitary–adrenal axis recovery.

**Conclusions:**

The preoperative 1 mg dexamethasone suppression test cortisol value and presence of diabetes are the only relevant predictor of hypothalamus–pituitary–adrenal axis recovery in patients with non-aldosterone- producing adrenocortical adenoma undergoing surgery, regardless other clinical and biochemical variables. Notably, pre- and postoperative ACTH levels did not predict hypothalamus–pituitary–adrenal axis recovery. These findings point towards the potential for saving resources by optimizing their allocation during follow-up assessments for patients with non-aldosterone-producing adrenocortical adenoma undergoing unilateral adrenalectomy.

## Introduction

In recent decades, the refinement and broader implementation of imaging techniques have led to an increased detection of adrenal incidentalomas (AIs), with current prevalence reaching about 2% in the adult population and 10% in individuals aged over 80 years [1]. The majority of these lesions are benign, predominantly adenomas originating from the adrenal cortex. Historically, according to the 2016 ESE-ENSAT guidelines, non-aldosterone-producing adrenocortical adenomas (NAPACAs) have been classified based on morning serum cortisol levels following 1 mg overnight dexamethasone suppression test (1 mg-DST) into three categories: autonomous cortisol secretion (ACS, where 1 mg-DST > 138 nmol/L), possible ACS (PACS, where 50 < 1 mg-DST ≤ 138 nmol/L), and non-functional adenoma (NFA, where 1 mg-DST ≤ 50 nmol/L) [2]. Nonetheless, emerging studies indicate that patients with cortisol-secreting adrenal adenomas face increased risks of morbidity and mortality regardless of the degree of hypercortisolism [3–10]. Therefore, the most recent 2023 guidelines have evolved beyond the PACS and ACS distinction, instead proposing the term “mild autonomous cortisol secretion” (MACS) [11].

Undoubtedly, lesions with clinically overt hormonal excess, such as primary aldosteronism, Cushing’s syndrome (CS), or pheochromocytoma, require surgical treatment [11]. Given the observed increase in cardiometabolic morbidity and mortality in patients with MACS [12–15], it stands to reason that the normalization of hypercortisolism, whether through medical treatment or surgery, should also be considered for these patients. However, current evidence supporting the use of adrenal steroidogenesis inhibitors in MACS is still scarce, and the latest ESE-ENSAT guidelines advocate for an individualized, center-specific approach when evaluating the need for surgical intervention in these patients [11]. Concerning surgical treatment, postoperative hypocortisolism (PH) is a common occurrence in patients with AI undergoing unilateral adrenalectomy. However, data regarding its prevalence and potential predictors are limited. Specifically, few studies have directly investigated potential associations between baseline clinical and biochemical characteristics and the subsequent development of PH in patients with AI. Emerging data on MACS and adrenal CS have highlighted a correlation between preoperative hypercortisolism severity, assessed through 1 mg-DST, and longer PH duration as well as the degree of preoperative hypothalamic–pituitary–adrenal (HPA) axis suppression, evaluated with baseline ACTH levels [16, 17]. Despite the absence of a definitive 1 mg-DST cutoff able to reliably predict the onset of PH [17–19], preliminary evidence has suggested that values below 1.2 μg/dL (33 nmol/L) might be predictive of post-surgical adrenal sufficiency [20] in NAPACAs. However, some authors argue against the use of 1 mg-DST alone in NAPACAs and adrenal CS, instead suggesting a multifactorial approach incorporating ACTH levels, urinary free cortisol (UFC), and midnight serum cortisol as more accurate in predicting PH [16–19]. Collectively, the complexities arising from the varying degrees of hypercortisolism, coupled with the lack of heterogeneity and the small sample size in previously published studies, complicates the identification of reliable predictors for PH.

Therefore, the aim of this study was to quantify the incidence of adrenal insufficiency and determine the timeframe for restoration of normal adrenal function in a homogeneous cohort of patients with NAPACA undergoing unilateral adrenalectomy and a subsequent, long-term endocrine workup. Furthermore, we sought to identify potential clinical and biochemical factors at baseline that could predict the onset of PH.

## Materials and methods

### Patient selection

This is a longitudinal observational retrospective analysis of consecutive patients aged 18–80 years, diagnosed with AI, originally included in the ITACA trial (NCT04127552), referred to the Department of Experimental Medicine and who underwent unilateral adrenalectomy at the Department of General Surgery, Surgical Specialties and Organ Transplantation “Paride Stefanini”, Sapienza University of Rome, from September 2019 to April 2023.

Only patients with NAPACA were included in the original study. Patients with a diagnosis of overt CS, primary aldosteronism, pheochromocytoma, adrenocortical carcinoma, adrenal metastasis, myelolipoma, congenital adrenal hyperplasia, and other rare pathologies were excluded, along with patients using drugs or affected by diseases known to impact on corticosteroid metabolism or cortisol secretion (i.e., suppressive doses of glucocorticoids, thyrotoxicosis, bowel diseases, chronic renal failure, chronic hepatic disease, depression, alcoholism, eating disorders, rheumatologic and hematological diseases). Adrenalectomy was performed after a multidisciplinary assessment, based on the presence of biochemically significant cortisol excess and/or on lesion size or growth rate, age and patient’s comorbidities.

Data on age, sex, anthropometric measures, tumor size, medical history, relevant comorbidities (overweight, obesity, glucose and lipid metabolism derangements, arterial hypertension, osteopenia, and osteoporosis) and tumor histology were collected from a review of medical records. Postoperative histological examination confirmed that all included lesions were consistent with adrenal cortex adenomas.

In accordance with the most recent ESE-ENSAT guidelines [11], all patients were stratified for cortisol secretion as follows: patients with 1 mg-DST above 50 nmol/L (> 1.8 mcg/dL) and without clinical features of CS were labeled as MACS, whereas those 1 mg-DST ≤ 50 nmol/L (≤ 1.8 mcg/dL) were classified as NFA.

The original study was approved by the Local Ethics Committee of Sapienza University in Rome (reference number 5279) and was conducted in accordance with the Declaration of Helsinki (1964) and its subsequent amendments. All enrolled patients provided their written informed consent to participate in the study.

### Assessment of HPA axis and perioperative procedures

Our department has an established endocrine workup for patients referred with AI.

HPA axis evaluation is performed before (hereafter baseline) and 6 days, 6 weeks, 6 months and 1 year after surgery in all patients. The decision for these specific time points was based on recommendations from guidelines, along with previous research showing that HPA axis recovery typically occurs within this time frame [11, 21–23].

For baseline assessments, collected data included morning serum cortisol, ACTH, dehydroepiandrosterone sulfate (DHEAS), 24-h UFC, and serum electrolytes (sodium and potassium), all measured between 8–9 AM after overnight fasting. Moreover, we collected results from the 1 mg-DST: serum cortisol levels measured at 8 AM after taking 1 mg of oral dexamethasone at 11 PM the night before.

All adrenalectomies were performed by a single surgeon (AMP) through laparotomic or laparoscopic approach, depending on the size of the adrenal adenoma and the patients’ clinical characteristics. In the absence of a universally accepted guideline for perioperative glucocorticoid management in adrenal surgery, our center has adopted a specific protocol. All patients, irrespective of NFA or MACS diagnosis, received a stress dose of hydrocortisone (100 mg IV) at the time of anesthesia induction. Postoperatively, NFA patients were closely monitored for 24 h with blood pressure and electrolyte assessments. In contrast, patients with MACS, in accordance with ESE-ENSAT guidelines [11], were treated with glucocorticoid replacement therapy post-surgery. This commenced with a dose of 50 mg IV every 8 h, transitioning to oral cortisone acetate (25–37.5 mg/day in divided doses) on the second postoperative day. All patients underwent a HPA axis assessment within day 6. Considering the short plasma half-life of cortisone acetate [24], HPA axis suppression due to exogenous glucocorticoids is unlikely and therefore HPA axis function was then assessed within 6 days through 8 AM cortisol levels, with replacement therapy discontinued at least 24 h before testing, according to previous research [25]. Patients with cortisol levels > 350 nmol/L (> 12.6 mg/dL) were considered to have adequate adrenal function and had their GC therapy stopped [22]. Those under this cutoff continued to receive standard replacement therapy until 6-week reassessment of the HPA axis through 250 μg short Synacthen (Sigma-tau Pharmaceutical) test. Patients failing the test at each assessment (6 weeks, 6 months, and 1 year) were reassessed with a short Synacthen test (SST) at the subsequent evaluation. The interpretation of the SST was based on the 30-min serum cortisol: namely, levels > 450 nmol/L (16.3 mcg/dL) defined the response as adequate [26].

### Laboratory analyses

All laboratory analyses were performed at the study center. Serum and 24-h urine cortisol was measured by radioimmunoassay, using Beckman Coulter reagents (ref. IM1841). The measurement range (from analytical sensitivity to the highest calibrator) is 8.60–2000 nM. The high specificity of the assay was confirmed by extremely low or undetectable cross-reactivity against other naturally occurring steroids (aldosterone, corticosterone, cortisone, 11-desoxycortisol, progesterone, etc.) or therapeutic drugs that may be present into patient samples (Prednisolone, Prednisone, Spironolactone, etc.). ACTH was measured by immunoradiometric assay, using Beckman Coulter reagents (ref. IM2030, B89463). EDTA plasma samples were collected and processed as soon as possible by centrifugation at 2–8 °C. Since ACTH is very unstable, plasma samples were stored at -18 °C unless the assay was done immediately. The measurement range (from analytical sensitivity to highest calibrator) is 0.31 to approximately 1500 pg/mL. The high specificity of the assay was confirmed by extremely low or undetectable cross-reactivity against related molecules (ACTH 1–24; 1–10; 18–39; 11–24; αMSH and POMC) either in the absence (cross-reactivities) or the presence (interferences) of ACTH. DHEAS was measured using a chemiluminescent microparticle immunoassay (CMIA), using ARCHITECT DHEAS reagent by Abbott (ref. 8K27). The measurement range is 3.0 μg/dL to 1500.0 μg/dL. The specificity of DHEAS assay is designed to have ≤ 10% cross-reactivity when tested with structurally similar compounds.

### Statistical analysis

Distribution of continuous variables was assessed with the Shapiro–Wilk test; linearity was established by visual inspection of a scatterplot. Categorical variables were expressed as percentage and frequency; continuous variables were reported as mean and SD or median and interquartile range (25–75th percentile) as appropriate per distribution of data. For group comparisons, unpaired Student’s T-test, Mann–Whitney, χ^2^ or Fisher’s exact test was used as appropriate. A binomial logistic regression was performed to ascertain the effects of selected variables on the likelihood that participants would show recovery at 6-week assessment. Linearity of the continuous variables with respect to the logit of the dependent variable was assessed via the Box–Tidwell procedure. A Bonferroni correction was applied in all the logistic regression models. Based on this assessment, all continuous independent variables were found to be linearly related to the logit of the dependent variable. A backward stepwise predictor selection was applied to the binomial logistic regression in order to begin with a full model and gradually eliminate any predictor that is not significant to the model. Multicollinearity among the predictor variables was identified by assessing the values of tolerance and VIF (Variance Inflation Factor). For the outcome variables significant at binomial logistic regression, receiver-operating characteristic (ROC) curve analysis was performed using a uniform threshold according to the 95% sensitivity on the ROC analysis. According to this, a cutoff of 1 mg-DST was identified and selected. Area under the curve (AUC) analysis was used to express the overall diagnostic accuracy of the index criterion. In order to evaluate the variation of ACTH levels over multiple postoperative timepoints, we conducted a repeated measures analysis. Due to the missing values, we employed a mixed-effects model using the Restricted Maximum Likelihood (REML) estimation method. This approach allowed us to account for both within-subject correlations and between-subject variability, while also handling the unbalanced nature of the data due to missing data in different timepoints. To investigate potential subgroup differences, we split the cohort according to the occurrence of HPA axis recovery at 6 weeks. A two-way mixed-effects model was, therefore, performed including the different timepoints (Baseline, day 6, 6 weeks, 6 months, and 1 year) as a within-subjects factor, and the recovery at 6 weeks (yes/no) as a between-subjects factor, to explore and compare the variation in ACTH levels over time in each subgroup. Subsequently, we conducted Holm–Sidak’s multiple comparisons test to explore specific differences between timepoints within each subgroup. A *p*-value < 0.05 was considered as a statistically significant difference. Statistical analyses were performed using SPSS, version 27 (IBM, Chicago, IL) and GraphPad Prism 7.0 software package (GraphPad Software, La Jolla, CA).

## Results

### Preoperative assessments

Among the 150 patients referred to our Department from September 2019 to April 2023 for AI, 51 underwent unilateral adrenalectomy; 19 were excluded due to presence of adrenal lesions that did not meet the inclusion criteria (overt Cushing’s syndrome, pheochromocytoma, primary aldosteronism, adrenocortical carcinoma, myelolipoma, adrenal metastasis and other rare pathologies), resulting in the final enrollment of 32 patients with NAPACA. The baseline characteristics of the cohort are summarized in Table 1. Nineteen patients (59.4%) were female, aged 49 to 71 years, all of whom were postmenopausal, none receiving hormone replacement therapy. Median age at baseline was 61 [51–66] years, and mean body mass index (BMI) was 27.5 ± 5.8 kg/m^2^. The mean lesion diameter was 42.3 ± 15.8 mm. Median level of 1 mg-DST was 87.4 [58–157] nmol/L. Preoperative HPA axis assessment showed a median ACTH level of 12.5 pg/mL [9.0–16.4]. The mean morning serum cortisol levels was 534.5 ± 188.6 nmol/L and mean UFC was 254.4 ± 149.1 nmol/24 h. Serum electrolytes were within the normal range in all patients.

**Table 1:** Baseline characteristics and comorbidities of the whole cohort and after stratifying for 1 mg-DST cortisol levels

	All (*N* = 32)	MACS (*N* = 25)	NFA (*N* = 7)	*p*-value
Age (years)	61 [51–66]	58 [49–66]	66 [64–67]	0.175
BMI (kg/m^2^)	27.5 ± 5.8	27.7 ± 6.4	26.6 ± 3.8	0.663
Female (%)	19 (59.4)	14 (56)	5 (71.4)	0.671
Size of the resected tumor (mm)	42.3 ± 15.8	39.9 ± 14.5	51.0 ± 18.25	0.099
1 mg-DST (nmol/L)	87.4 [58.0–157.0]	110.5 [73.1–262.5]	35.2 [33.0–44.0]	**< 0.001**
ACTH (pg/mL)	12.5 [9.0–16.4]	12.0 [8.8–15.2]	32.1 [11.9–45.7]	0.061
Cortisol (nmol/L)	534.5 ± 188.6	554.6 ± 189.2	426.72 ± 181.3	0.261
UFC (nmol/24 h)	254.4 ± 149.1	257.3 ± 158.1	237.7 ± 103.6	0.840
DHEAS (µg/dL)	39.9 [14.9–81.4]	34.7 [20.9–71.1]	91.3 [15.1–164.7]	0.413
SBP (mmHg)	135 [130–140]	140 [130–140]	130 [120—135]	0.190
DBP (mmHg)	80 [75—85]	80 [80—85]	70 [70—80]	**0.023**
Sodium (mmol/L)	142 [141–144]	143 [141—144]	141 [141—143]	0.420
Potassium (mmol/L)	4.2 [4.1–4.5]	4.3 [4.1–4.7]	4.2 [4.1–4.3]	0.370
Overweight (%)	11 (34.4)	8 (32)	3 (42.9)	0.667
Obesity (%)	8 (25)	7 (28)	1 (14.3)	0.646
Type 2 diabetes (%)	3 (9.4)	2 (8)	1 (14.3)	0.536
IFG or IGT or IR (%)	9 (28.1)	6 (24)	3 (42.9)	0.370
Arterial hypertension (%)	23 (71.9)	18 (72)	5 (71.4)	1.000
Dyslipidemia (%)	15 (46.9)	11 (44)	4 (57.1)	0.678
Osteoporosis (%)	8 (25)	7 (28)	1 (14.3)	0.646
Osteopenia (%)	11 (34.4)	10 (40)	1 (14.3)	0.374

Continuous data are expressed as mean ± SD or median [IQR], as appropriate. Categorical variables are expressed as frequency (%). 1 mg-DST, 1 mg dexamethasone suppression test; *ACTH* adrenocorticotroph hormone; *BMI* body mass index; *DBP* diastolic blood pressure; *DHEAS* dehydroepiandrosterone sulfate; *SBP* systolic blood pressure; *UFC*, 24 h urinary free cortisol; *IFG*, impaired fasting glucose; *IGT*, impaired glucose tolerance; *IR* insulin-resistance

Significant *p*-values are highlighted in bold

Regarding the assessment of the main comorbidities at baseline, arterial hypertension, dyslipidemia, osteoporosis, and osteopenia were found in 71.9%, 46.9%, 25% and 34.4% of patients, respectively. Twelve (37.5%) patients had glucose metabolism impairment, of whom 3 (9.4%) had type 2 diabetes mellitus and 9 (28.1%) were diagnosed with either impaired fasting glucose, impaired glucose tolerance or insulin-resistance. Eleven (25%) patients were overweight and 8 (25%) were affected by obesity. According to 1 mg-DST, 7 patients (21.8%) had a diagnosis of NFA and 25 (78.2%) patients had MACS. Both groups were similar in terms of sex distribution, age, and BMI. There were no differences in the prevalence of the main comorbidities between MACS and NFA. As expected, 1 mg-DST was significantly higher in MACS compared to NFA (median DST 110.5 [73.1–262.5] nmol/L *vs* 35.2 [33.0–44.0], p < 0.001), as was diastolic blood pressure (*p* = 0.023). The clinical and biochemical characteristics of all patients, stratified into MACS and NFA, are summarized in Table 1.

### Univariate analysis

**Fig. 1:**
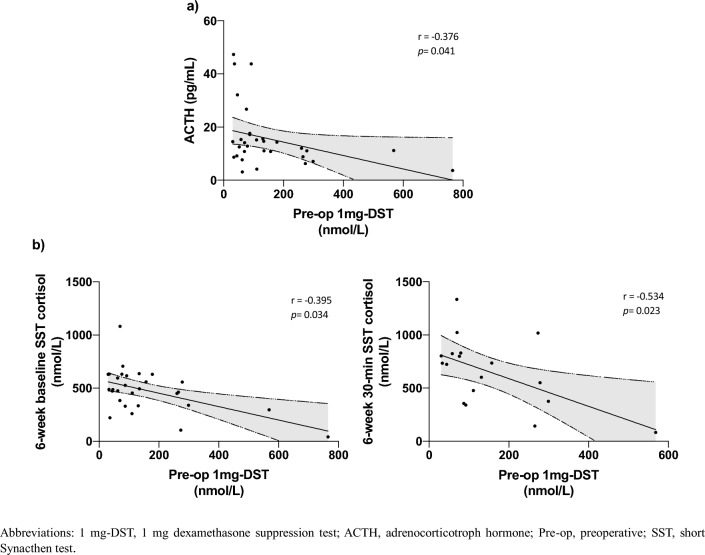
Univariate analysis of biochemical parameters in 32 patients with non-aldosterone-producing adrenocortical adenomas at baseline (**a**) and 6 weeks after unilateral adrenalectomy, (**b**). 1 mg-DST, 1 mg dexamethasone suppression test; *ACTH* adrenocorticotroph hormone; *Pre-op* preoperative; *SST* short Synacthen test

Cortisol levels after 1 mg-DST showed a negative correlation with baseline ACTH levels (*r* = − 0.376; *p* = 0.041) (Fig. 1a). Furthermore, 1 mg-DST was also negatively associated with the 6-week baseline (*r* = − 0.395, *p* = 0.034) and 30-min cortisol levels during SST (*r* = − 0.534, *p* = 0.023) (Fig. 1b). None of the patients’ comorbidities was associated with postoperative HPA axis function.

### HPA axis recovery

Six days after adrenalectomy, 6 patients (18.8%, 4 NFA and 2 MACS) presented with normal adrenal function, whereas 26 (81.2%, 3 NFA and 23 MACS) had morning cortisol levels consistent with adrenal insufficiency.

At the 6-week postoperative timepoint, 14 of the 26 patients (53.8%, 3 with NFA and 11 with MACS) recovered adrenal function. No difference in age, sex, BMI, tumor size, ACTH, serum cortisol, UFC, DHEAS, blood pressure, electrolytes were found between patients recovering compared to those still facing adrenal insufficiency. However, the former had lower 1 mg-DST compared to the latter (median 1 mg-DST 76.2 [61.8–111.0] nmol/L *vs* 260.0 [113.0–288.5] nmol/L, *p* < 0.001) (Table 2).

**Table 2:** Baseline characteristics of 26 patients with postoperative hypocortisolism, stratified for recovery at 6 weeks after adrenalectomy

	Recovery within 6 weeks (*n* = 14)	No recovery at 6 weeks (*n* = 12)	*p*-value
Age (years)	61 [57–66]	52 [41–67]	0.131
BMI (kg/m^2^)	29.8 ± 4.9	26.5 ± 6.8	0.166
Female (%)	8 (57.1)	7 (58.3)	0.951
Lesion size (mm)	40.8 ± 13.2	41.6 ± 20.1	0.904
1 mg-DST (nmol/L)	76.2 [61.8–111.0]	260.0 [113.0–288.5]	** < 0.001**
ACTH (pg/mL)	14.3 [10.8–15.6]	11.2 [7.9–13.6]	0.252
Cortisol (nmol/L)	533.6 ± 219.9	512.1 ± 181.0	0.789
UFC (nmol/24 h)	324.7 ± 166.8	206.5 ± 114.8	0.114
DHEAS (µg/dL)	69.8 [29.7–80.4]	30.6 [14.5–67.1]	0.494
SBP (mmHg)	130 [120—140]	140 [130–140]	0.320
DBP (mmHg)	80 [70–85]	80 [80–87]	0.574
Sodium (mmol/L)	142 [141–144]	143 [142–144]	0.494
Potassium (mmol/L)	4.3 [4.2–4.6]	4.2 [3.9–4.5]	0.297

Continuous data are expressed as mean ± SD or median [IQR], as appropriate. Categorical variables are expressed as frequency (%)

1 mg-DST, 1 mg dexamethasone suppression test; *ACTH* adrenocorticotroph hormone; *BMI*, body mass index; *DBP* diastolic blood pressure; *DHEAS* dehydroepiandrosterone sulfate; *SBP* systolic blood pressure; *UFC* urinary free cortisol

Significant *p*-values are highlighted in bold

**Fig. 2:**
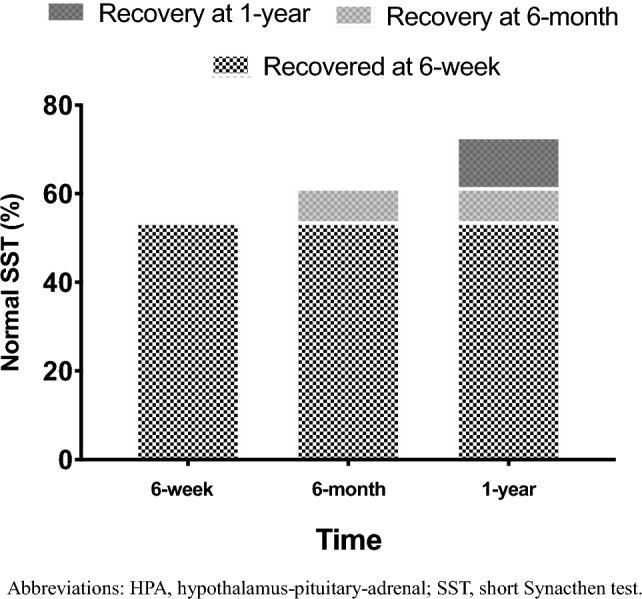
Percentage of HPA axis recovery among 26 patients with non-aldosterone-producing adrenocortical adenomas undergoing unilateral adrenalectomy and exhibiting postoperative adrenal insufficiency assessed within 6 days. *HPA* hypothalamus–pituitary–adrenal; *SST* short Synacthen test

Among the 12 patients (all with MACS) who still exhibited adrenal insufficiency at the 6-week evaluation, 2 (16.6%) and 3 (25%) recovered 6 months and 1 year after surgery, respectively. At the end of the study, 7 (26.9%) patients had yet to recover HPA axis function. Percentage of recovery among the 26 patients with adrenal insufficiency at within day 6 is shown in Fig. 2.

### Predictors of HPA axis recovery

A backward binomial logistic regression was performed to examine the ability of preoperative biochemical assessments and comorbidities (1 mg-DST, ACTH, serum cortisol, tumor size, BMI, hypertension, diabetes, and osteoporosis) to predict HPA axis recovery at 6 weeks from adrenalectomy. The model was statistically significant (*χ*^2^ = 17.719; *p* < 0.001) and explained 61% of the variance in HPA recovery within the 6-week assessment, correctly classifying 83.3% of cases. Sensitivity was 89.5%, specificity was 72.7%, positive predictive value was 85%, and negative predictive value was 80%. Among all the variables incorporated into the model, only 1 mg-DST and presence of diabetes at the time of the test were statistically significant. Higher preoperative 1 mg-DST levels (OR = 1.02, *p* = 0.008) and diagnosis of diabetes at the time of the test (OR = 24.55, *p* = 0.036) were independent predictors for reduced likelihood of HPA axis recovery within 6 weeks. Detailed description for all coefficients in the multiple regression analysis is shown in Table 3. When preoperative diagnoses consistent with NFA were excluded from the analysis, the results did not change (data not shown).

**Table 3:** Backward stepwise predictor selection applied to logistic regression model in 32 patients with non-aldosterone-producing adrenocortical adenomas treated with adrenalectomy

	*χ*^2^ = 17.719. *p* < 0.001	Dependent: 6-week HPA axis recovery			
	Independent	OR	95% CI lower bound	95% CI upper bound	*p*-value
Step 1	1 mg-DST	1.03	1.002	1.067	**0.036**
	ACTH	1.03	0.842	1.259	0.774
	Serum cortisol	1.00	0.994	1.001	0.569
	Tumor size	1.05	0.915	1.198	0.507
	Hypertension	17.54	0.238	1000.000	0.192
	Diabetes	75.44	0.178	31,949.115	0.161
	Osteoporosis	11.20	0.206	608.184	0.236
	BMI	1.27	0.910	1.761	0.162
Step 7	1 mg-DST	1.02	1.005	1.036	**0.008**
	Diabetes	24.55	1.239	486.322	**0.036**

Significant *p*-values are highlighted in bold

**Fig. 3:**
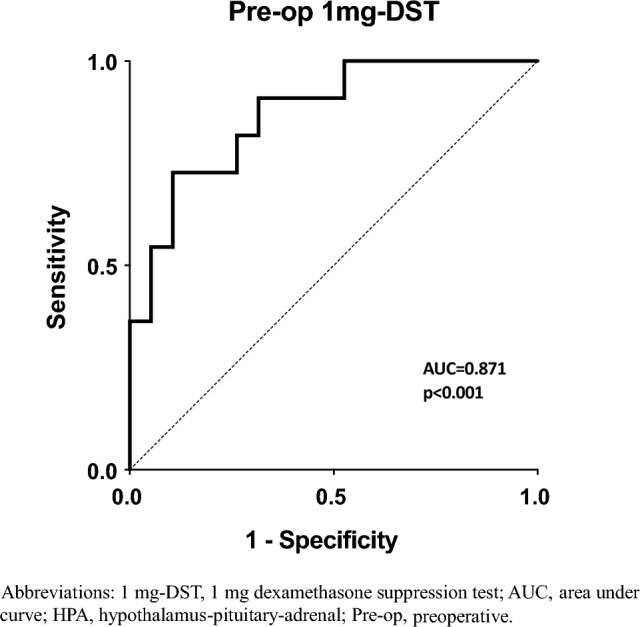
ROC curve analysis to determine the ability of the preoperative cortisol levels at 1 mg-DST to predict HPA axis function 6 weeks after adrenalectomy. 1 mg-DST, 1 mg dexamethasone suppression test; *AUC* area under curve; *HPA* hypothalamus–pituitary–adrenal; *Pre-op* preoperative

According to the ROC analysis, a preoperative 1 mg-DST level ≤ 131 nmol/L (≤ 4.7 mcg/dL, ROC AUC 0.87; 95% CI 66.9–98.7, *p* < 0.001) best predicted HPA axis recovery at 6 weeks from adrenalectomy, with a sensitivity of 89.5% and a specificity of 72.7% (Fig. 3). When stratified according to the mentioned cutoff, the prevalence of HPA axis recovery at 6 weeks was higher in patients below that threshold when compared to those above (78.6% vs 21.4%, *χ*^2^ = 9.726, *p* = 0.04), giving a 14 times higher chance to recovery in the former.

### Temporal dynamics and predictive value of ACTH levels following unilateral adrenalectomy

**Fig. 4:**
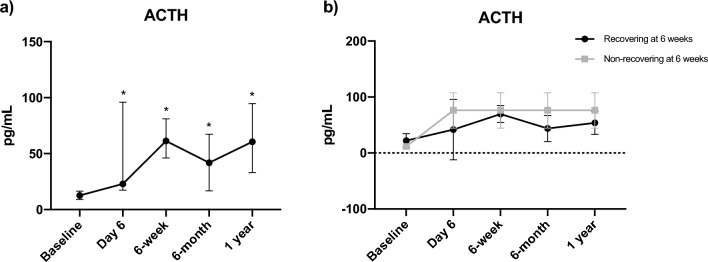
Variations of ACTH levels in 32 patients undergoing unilateral adrenalectomy for non-aldosterone-producing adrenocortical adenomas (**a**), stratified according to the occurrence of HPA axis recovery at 6 weeks (**b**). *p < 0.05 *vs* baseline

ACTH levels did not emerge as predictive of adrenal recovery at 6 weeks in the logistic regression analysis (Table 3). To better understand the dynamic variations of ACTH over time, and to identify any potential patterns predictive of recovery, we carried out a mixed-effects analysis. This analysis unveiled significant differences in ACTH levels across timepoints (*F* = 6.692, *p* < 0.001). Post hoc comparisons employing Holm–Sidak’s test identified significant differences between baseline and all postoperative ACTH levels, though no differences emerged between timepoints following unilateral adrenalectomy. When comparing baseline measurements to various postoperative intervals, we recognized a specific pattern. ACTH significantly increased by day 6, with a predicted (LS) mean difference of 50.06 pg/mL (95% CI 5.92–94.20, *p* = 0.018) but then plateaued, with mean differences maintaining consistent levels of 53.24 pg/mL (95% CI 19.86–86.63, *p* < 0.001 vs baseline), 44.65 pg/mL (95% CI 5.72–83.59, *p* = 0.017 vs baseline), and 47.07 pg/mL (95% CI 11.91–82.24, *p* = 0.003 vs baseline) at the 6-week, 6-month, and 1-year marks, respectively (Fig. 4a). In addition, to explore potential differences in ACTH levels variations between patients recovering within 6-week postoperative and those who did not, we conducted a two-way mixed-effects analysis. However, this did not yield significant results, suggesting that the observed changes in ACTH levels over time were not affected by the recovery status within the first 6 weeks after adrenalectomy (Fig. 4b).

## Discussion

Our study underscores the value of the 1 mg-DST in predicting the likelihood of HPA axis recovery following unilateral adrenalectomy in patients with NAPACA. We also detected that certain parameters, including preoperative ACTH levels, may not be useful in stratifying patients who develop PH. Furthermore, the HPA axis shows signs of recovery as early as six weeks postoperative, emphasizing the necessity for an early assessment in order to potentially avoid needless glucocorticoid replacement therapy in patients who are on the path to restoring adrenal function.

In our cohort, PH was observed in over 80% of patients. Our results align well with previous studies, in which the average prevalence of PH in patients undergoing unilateral adrenalectomy for AI often exceeds 50% [16–23, 27]. Only a few reports have observed lower average percentages of patients developing PH [19, 28, 29]. However, the disparity among studies could be attributed to variations in the diagnostic criteria and cutoffs used to define PH. In addition, the inclusion criteria for many studies extend to patients with overt CS, which may further contribute to the variability observed. There is currently no established consensus regarding the ideal timeline for reassessing patients' HPA axis recovery after adrenalectomy. Interestingly, from 6 weeks onward, an additional 23% of our patients demonstrated adrenal function recovery within 1 year. However, due to the limited sample size, we were unable to identify specific clinical or biochemical predictors that might influence the recovery timeline in these patients.

Our study confirmed previous findings regarding the relationship between cortisol levels after a 1 mg-DST and baseline HPA suppression (for instance, ACTH levels), while also providing significant insights into predicting 6-week HPA function outcomes (baseline and 30-min SST cortisol levels). Specifically, our logistic regression analysis demonstrated that the degree of hypercortisolism, as measured by the 1 mg-DST, is indeed the only reliable biochemical predictor of PH in patients with NAPACA. Notably, a serum cortisol concentration of over 131 nmol/L post 1 mg-DST emerges as the strongest biochemical indicator of prolonged postoperative HPA axis recovery. Conversely, patients with preoperative levels less than or equal to 131 nmol/L typically experienced an HPA axis recovery period of around 6 weeks. In other terms, any increase of 10 nmol/L in 1 mg-DST levels confers a 21% reduction in the probability of recovering HPA axis within 6 weeks. There is still disagreement in the literature regarding the cutoffs used to determine HPA axis impairment in NAPACAs. For instance, some authors prefer a serum cortisol value greater than 50 nmol/L after a 1 mg-DST, in line with recommendations from the European Society of Endocrinology [11, 16], or slightly higher cutoffs, such as 83 nmol/L [18; 19]. Conversely, Hurtado et al. found a significantly higher cutoff value for serum cortisol after a 1 mg-DST (> 276 nmol/L) [17]. However, this retrospective study in a mixed cohort of patients with adrenal CS and MACS undergoing adrenalectomy, examined the interval of HPA axis recovery looking at baseline clinical and biochemical variables as parameters influencing the duration and dosage of glucocorticoid replacement therapy. They identified the degree of 1 mg-DST, age at diagnosis, and duration of symptoms before diagnosis as the strongest predictors of postoperative glucocorticoid therapy duration.

However, despite the prediction of postoperative adrenal insufficiency often required a combination of multiple biochemical parameters in previous studies, our results demonstrated that, besides the 1 mg-DST, no other biochemical parameter predicted HPA axis recovery. This suggests that certain traditional measures might not always offer the most accurate insight into patient recovery and underscores the need for continued exploration of alternative predictors. The disparity in the 1 mg-DST cutoff values between our study and others could be attributed to our more precise patient selection; we exclusively included patients with NAPACAs, thereby excluding overt CS. Regarding symptom duration, while it is clear that the onset of symptoms in incidentally discovered adrenal masses, in terms of cortisol-related comorbidity, is associated with the likelihood of patients being linked to medical care, this association can be influenced by numerous confounding factors, including ease of access to care and socioeconomic variables. Although many comorbidities, such as diabetes, hypertension, and low bone mineral density, are non-specific and highly prevalent in the general population, our results revealed that the preoperative presence of diabetes was related to postoperative HPA function. Our results show that patients diagnosed with diabetes at the time of the test have 24 times higher chance of failing the Synacthen test at 6 weeks. There is a growing body of evidence on the association between MACS and hyperglycaemia to the extent that the ESE-ENSAT guidelines recommend screening for diabetes in patients with adrenal incidentaloma and MACS [11]. Several studies demonstrate that diabetes mellitus is linked to a heightened risk of postoperative complications [30]. Although the impact of cortisol-related comorbidities on postoperative HPA axis recovery have not yet been fully investigated, our findings align with a prior study that observed an elevated occurrence of diabetes in correlation with reduced postoperative cortisol levels among patients undergoing adrenalectomy for hypercortisolism [31].

Of note, preoperative ACTH levels failed to predict the likelihood of HPA axis recovery postoperative. Recent guidelines advocate the demonstration of ACTH-independency, evidenced by suppressed or low morning plasma ACTH levels at baseline, before considering adrenalectomy in MACS [11]. Yet, its use as a complementary test to 1 mg-DST in diagnosing MACS is not without limitations. Olsen et al. found suppressed basal ACTH in 19% of patients with NFA compared with 4% of control subjects [32]. The same research group suggested using ACTH levels after a 1 mg-DST as a marker of HPA axis suppression in MACS. They proposed that a 1 mg-DST greater than 50 nmol/L, coupled with either ACTH levels after 1 mg-DST of less than 0.6 pmol/L or an ACTH-ratio (defined as ACTH after 1 mg-DST/basal ACTH) less than 18%, should raise suspicion of MACS [33]. In our study, we found no difference in basal ACTH levels or other evaluation timepoints within a year between patients who recovered within 6 weeks and those who still exhibited adrenal insufficiency at the six-week assessment. While it is essential to demonstrate ACTH-independency in the hypercortisolism workup, our findings underscore the limited utility of ACTH values in the follow-up assessments of patients with MACS.

Moreover, as expected, unilateral adrenalectomy led to significant changes in ACTH levels over time. However, beyond an early surge in ACTH on day six, levels plateaued, with no further significant changes observed over a one-year period, irrespective of patient recovery status. Existing literature reports high inter-assay variability and low reproducibility of plasma ACTH assays between studies [34, 35]. One study found only 85% of all ACTH measurements were interpreted correctly, and this dropped to 60% in patients with suspected ACTH-independent CS [36]. In another study, detectable plasma ACTH concentrations were reported in up to 60% of patients with adrenal CS [37]. Hence, plasma ACTH values must be interpreted with caution due to the wide patient result variation, which often complicates the differentiation between ACTH-dependent and ACTH-independent CS. Even with modern ACTH assays, ACTH levels can deteriorate quickly in whole blood unless cooled and separated, and the plasma rapidly frozen until needed. This underscores the significant healthcare costs associated with the care of patients with MACS and challenges the usefulness of ACTH measurement in a postoperative setting.

While this study has provided meaningful insights, it is worth noting its limitations. As this analysis was retrospective in nature and relied on data obtained from electronic patient records, it may have been subject to potential selection bias, particularly in those individuals undergoing HPA axis testing. Even though we demonstrated that the 1 mg-DST can be used as a predictor of recovery, the cutoff values derived from our dataset need validation through prospective studies in larger cohorts before they can be confidently applied in clinical practice. This will contribute towards the development of standardized algorithms to predict HPA axis recovery and optimize the utilization of healthcare resources. Despite these limitations, the study presents important strengths. It includes a 1-year follow-up after adrenalectomy, which provides a comprehensive overview of patient recovery. In addition, all endocrinological assessments were conducted by a consistent team of clinicians, adhering to a uniform protocol for diagnosis, management, and follow-up. This consistency enhances the reliability of our findings.

## Conclusions

The management of PH in patients undergoing adrenalectomy needs an individualized approach, which should take into account preoperative clinical and biochemical factors. However, our study shows that in patients with NAPACA, the 1 mg-DST is the only reliable biochemical predictor of HPA axis recovery, highlighting the pivotal role that the severity of hypercortisolism plays in shaping the duration of PH. Furthermore, our study suggests that neither tumor size, preoperative or postoperative ACTH levels provide useful insights into predicting future adrenal function. Thus, ACTH levels should not be considered as a marker of HPA axis recovery and should not be routinely assessed in patients undergoing unilateral adrenalectomy. These findings underscore the intricate dynamics of the HPA axis postoperative and provide valuable insights into its temporal evolution. Further prospective studies with larger cohorts are needed to fully elucidate the natural history of postoperative adrenal insufficiency in patients with AI.

## Data Availability

The datasets generated during and/or analyzed during the current study are available from the corresponding author on reasonable request.
